# G Protein–Coupled Estrogen Receptor 30 Reduces Transverse Aortic Constriction–Induced Myocardial Fibrosis in Aged Female Mice by Inhibiting the ERK1/2 -MMP-9 Signaling Pathway

**DOI:** 10.3389/fphar.2021.731609

**Published:** 2021-11-05

**Authors:** Xiaowu Wang, Jipeng Ma, Shuaishuai Zhang, Zilin Li, Ziwei Hong, Liqing Jiang, Weixun Duan, Jincheng Liu

**Affiliations:** Department of Cardiovascular Surgery, Xijing Hospital, Fourth Military Medical University, Xi’an, China

**Keywords:** G protein–coupled estrogen receptor 30 (GPR30), transverse aortic constriction, MMP-9, aged female, cardiac fibrosis

## Abstract

The incidence of cardiovascular diseases was significantly increased in postmenopausal women. The protection of estrogen in the cardiovascular system has been further reported for decades. Although menopausal hormone therapy has been used in many clinical trials, the debatable results indicate that the studies for elucidating the precise molecular mechanism are urgently required. G protein–coupled estrogen receptor 30 (GPR30) is a membrane receptor of estrogen and displays protective roles in diverse cardiovascular diseases. Previous studies have revealed that ERK1/2-mediated MMP-9 signaling was involved in ischemic heart diseases. However, the role of ERK1/2-mediated MMP-9 signaling in the protection of GPR30 against cardiac hypertrophy in aged female mice has not been investigated. Our present study demonstrated that GPR30 overexpression and its agonist G1 co-administration reduced transverse aortic constriction–induced myocardial fibrosis and preserved cardiac function in aged female mice. MMP-9 expression was markedly increased *via* ERK1/2 phosphorylation in transverse aortic constriction–injured myocardium of aged female mice. Further results showed that GPR30/G1 activation decreased MMP-9 expression *via* ERK1/2 inhibition, which further reduced TGF-β1 expression. Inhibition of the ERK1/2 signaling pathway by its inhibitor PD98059 suppressed the induction of the cardiomyocyte MMP-9 level caused by the GRP30 antagonist G15 and inhibited TGF-β1 expression in cardiac fibroblast *in vitro*. In summary, our results from *in vivo* and *in vitro* studies indicated that GPR30 activation inhibited myocardial fibrosis and preserved cardiac function *via* inhibiting ERK-mediated MMP-9 expression. Thus, the present study may provide the novel drug targets for prevention and treatment of cardiac pathological hypertrophy in postmenopausal women.

## Introduction

Cardiovascular disease is the leading cause of death worldwide, which accounts for 17.3 million death each year (Mendis et al., 2011; Laslett et al., 2012). Clinical studies revealed that the incidence of cardiovascular diseases was much lower in premenopausal women than in age-matched men (Iorga et al., 2017), whereas women at age of 50 years have high cardiovascular risk as compared to men at age of 70 years (Lloyd-Jones et al., 2006). These studies strongly indicate that estrogen and its receptor-mediated signaling pathways may play a cardioprotective role in diverse cardiovascular diseases in postmenopausal women (Aryan et al., 2020). Furthermore, a clinical statistical analysis showed that patients who had bilateral oophorectomy were associated with double risk of coronary heart disease, as compared with age-matched premenopausal women (Parker et al., 2009). Although accumulating evidence suggested the increased cardiovascular risk after menopause, menopausal hormone therapy (MHT) for prevention and treatment of cardiovascular diseases is still controversial (Schierbeck et al., 2012; Boardman et al., 2015; Manson et al., 2020). Thus, elucidating the underlying molecular mechanisms may identify the potential drug targets for treating cardiovascular diseases in postmenopausal women.

G protein–coupled receptor 30 (GPR30), also designated as G protein–coupled estrogen receptor (GPER), is a seven-transmembrane protein and a novel estrogen receptor, besides the two classic nuclear receptors ERα and ERβ. Previous studies have demonstrated that GPR30 is widely expressed and participates in multiple biological functions including glucose tolerance, bone growth, and blood pressure regulation (Chagin & Sävendahl, 2007; Hazell et al., 2009; Lindsey et al., 2009; Mårtensson et al., 2009; Groban et al., 2019). It was further revealed that GPR30 activation protected neurons from ischemia-induced injury through autophagy regulation (Wang et al., 2020). GPR30 preserved neuronal survival in a cerebral ischemia mouse model induced by cardiac arrest and cardiopulmonary resuscitation (Kosaka et al., 2012). In particular, our previous data showed that GPR30 protected the heart against myocardial infarction and diabetes-induced cardiac dysfunction in the female ovariectomized murine model (Wang et al., 2018; Wang et al., 2019). However, the role of GPR30 in the hypertrophied heart of aged female mice needs further investigation.

MMP-9 belongs to matrix metalloproteinase superfamily and can maintain extracellular matrix homeostasis *via* matrix metalloprotein degradation (Radosinska et al., 2017). It thus exacerbated cardiac fibrosis and inhibited angiogenesis following myocardial infarction since extracellular matrix remodeling was a key pathological feature of ischemia cardiac injury (Ducharme et al., 2000; Lindsey et al., 2006). However, whether GPR30 activation could regulate MMP-9 expression was still unclear. Therefore, the present study was designed to investigate the role of GPR30, and its downstream signaling pathway focusing on MMP-9–mediated fibrosis in transverse aortic constriction (TAC)–induced cardiac hypertrophy of aged female mice.

## Materials and Methods

### Reagents

GPR30 (ab37742), vimentin (ab92547), cardiac troponin I (ab47003), TGF-β1 (ab27969), and MMP-9 38,898) were purchased from Abcam. ERK 4,695) and *p*-ERK (4,370) were purchased from Cell Signaling Technology. GAPDH was obtained from Santa Cruz Biotechnology (Santa Cruz, CA, United States ). The rabbit anti-goat, goat anti-rabbit, and goat anti-mouse secondary antibodies were purchased from the Zhongshan Company (Beijing, China). The MMP-9 ELISA kit was purchased from Elabscience Bioengineering Institute (Wuhan, China). GPR30 agonist G1 (10,008,933) and GPR30 antagonist G15 14,673) were purchased from Cayman Chemical (Ann Arbor, MI, United States ). The ERK inhibitor PD98059 (HY-12028) and DOX (HY-15142) were purchased from MCE. AAV-GPR30 was purchased from the GeneChem Company (Shanghai, China). DAPI was obtained from Roche Molecular Biochemicals (Bayer, Mannheim, Germany). The ECL reagent was purchased from Millipore (Billerica, MA, United States ).

### Experimental Animals

Female C57BL/6 mice (20–25 g, 18 months old, obtained from the Experimental Animal Center of Fourth Military Medical University) were housed in individual cages under a 12:12-h light/dark cycle (light on 06:00) at 22–24 °C and fed with regular pellet diet *ad libitum*. The subjects in this study were mice which were raised and used in experiments in accordance with the Guide for the Care and Use of Laboratory Animals of the Chinese Animal Welfare Committee.

### *In vivo* Experimental Design and Treatment

All animal experiments were approved by the Institutional Animal Care and Use Committee of Fourth Military Medical University. The *in vivo* experiments were designed to investigate the influence of the estrogen receptor GPR30 on the cardiac function and myocardial fibrosis in hypertrophied aged female mice. Mice were randomly divided into the following four experimental groups: 1) aged female mice (AG group), 2) the AG + GPR30/G1 group, 3) the AG/TAC group, and 4) the AG/TAC + GPR30/G1 group. Mice were subjected to a tail intravenous injection of 5× 10^10^ adeno-associated virus genome particles carrying GPR30 in the AG + GPR30/G1 group and the AG/TAC + GPR30/G1 group, while the equal amount of empty adeno-associated virus was injected in the AG group and the AG/TAC group. Briefly, awake mice were placed in a special device to avoid the movement of mice for tail vein injection. Then the tail of the mouse was wiped with a 75% ethanol cotton ball, followed by a tail vein injection with the volume of 50 μl. Mice in the AG/TAC group and the AG/TAC + GPR30/G1 group underwent TAC operation after 3 weeks, while those in the AG group and the AG + GPR30/G1 group were underwent sham operation. Following the surgery, the AG + GPR30/G1 group and the AG/TAC + GPR30/G1 group were injected intraperitoneally with G1 each day with a dose of 35 μg/kg body weight. The cardiac function of the mouse from each group was assessed at the time of fourth and eighth weeks following the operation.

### Transverse Aortic Constriction Surgery Protocol

TAC surgery was performed to establish cardiac hypertrophy in the mouse model, as previously described (Wang et al., 2013). Briefly, mice were anesthetized in an induction chamber with 1–2% isoflurane mixed with pure oxygen (0.5–1.0 l/min), which were then intubated endotracheally and connected to a ventilator (Minivent Type 845, Hugo Sachs Electronic, March, Germany, 100–120/min 0.15-ml tidal volume). Median thoracotomy was performed to expose the aortic arch, which was then constricted using a 7–0 silk suture tied firmly with a 27-gauge needle between the carotid arteries. The needle was immediately removed, and the chest was closed and sutured. Sham-operated mice underwent the same operation procedure, except for the ligation of aortic arch. After the surgery, mice were placed on a 38°C insulation blanket to keep warm and were observed carefully until they were free to move around.

### Echocardiography

The ultrasound technicians were not informed of the protocol of the study and the details of the animal groups to ensure unbiased reporting. After the mouse was anesthetized with 1–2% isoflurane, the left front chest hair of the mouse was removed. Transthoracic ultrasonography was performed using a VisualSonics 2,100 echocardiograph (FUJIFILM VisualSonics, Toronto, ON, Canada), and a 30-MHz transducer was used to obtain the images in both parasternal long axis and short axis of the left ventricle. The indexes that can be detected through echocardiography included ejection fraction (EF)% and fraction shortening (FS)% (indicating the cardiac function), interventricular septal thickness at end diastole (IVSd), and left ventricular posterior wall thickness at end diastole (LVPWd) (indicating the thickness of ventricular wall). The aforementioned parameters were calculated by Vevo Lab 3.1.0 software (FUJIFILM VisualSonics. Toronto, ON, Canada).

### Heart Weight, Body Weight, and Histological Examination

After completion of the experiments, mice were euthanized, and their hearts were harvested. The heart weight (HW) and body weight (BW) were recorded. From these data, the HW/BW ratios were calculated.

Hearts of mice used for morphological observation were fixed in 4% paraformaldehyde for 72 h, embedded in paraffin, and cut into 5-μm sections. Histomorphology was evaluated by hematoxylin and eosin (H&E) staining. Myocardial fibrosis was evaluated by Masson Staining, and the cell cross-sectional area was evaluated by wheat germ agglutinin (WGA) staining.

### Neonatal Rat Cardiomyocytes and Neonatal Rat Cardiac Fibroblasts Culture and Treatment

Newborn Sprague–Dawley (SD) rats were obtained from the Experimental Animal Center of the Fourth Military Medical University. Isolation and culture of neonatal rat cardiomyocytes (NRCMs) and neonatal rat cardiac fibroblasts (NRCFs) were performed, as described previously (Zhai et al., 2017). Briefly, the heart was harvested from newborn SD rats and cut into small pieces. The small pieces of myocardium were digested in phosphate-buffered saline (PBS) solution containing 1% collagenase I (Sigma V900891, Sigma-Aldrich, St. Louis, MO, United States). After passing through the sieve to remove tissue fragments, the cells were culture for 2 h in a CO_2_ incubator. The non-adherent NRCMs were transferred to another culture flask or confocal dish, and the remaining adherent cells are mostly NRCFs. NRCMs were plated at a density of 5× 10^5^ cells per ml and were cultured in the serum containing culture medium DME/F-12 (Gibco, Carlsbad, CA, United States ), 10% new bovine serum (Gibco, Carlsbad, CA, United States ), penicillin (100 U/ml), streptomycin (100 U/ml), and bromodeoxyuridine (BrdU) (0.1 mM) for 48 h at 37 °C with 5% CO_2_. After culturing for 24 h, NRCFs were plated at a density of 5×10^5^ cells/ml and cultured in the same medium as NRCMs.

*In vitro* experiments are designed to investigate the expression of *p*-ERK1/2, ERK1/2, and MMP-9 in primary cultured cardiomyocytes in the Dox + Ang Ⅱ state (simulating the aging pressure-overload stress) by the GPR30 agonist G1/antagonist G15 and the changes in the content of MMP-9 in the culture medium of each group. After the intervention of G1 or G15, the effects of the culture supernatant on cardiomyocytes and the subsequent different treatments on the expression of TGF-β1 of cardiac fibroblast were examined. The neonatal rat cardiomyocytes were isolated and divided into five groups. The cell without any treatment was considered as the control group (CON group). The cells in the Dox group were treated with 0.1 μM Dox for 24 h to mimic cell senescence, while the cells in the Dox + Ang II group were treated with 0.1 μM Dox combined with 0.1 μM Ang II for 24 h (Spallarossa et al., 2009). Cardiomyocytes were then treated with GPR30 agonist 10 nM G1 or GPR30 antagonist 1 μM G15 for 24 h in the indicated groups. For inhibition of ERK1/2 by its inhibitor, cells were first treated with 10 μM PD98059 for 24 h. The culture medium following diverse treatments was then transferred to cardiac fibroblast for 24-h treatment to examine the interaction between cardiomyocytes and fibroblast.

### Immunofluorescence Staining

The immunofluorescence staining experiments were performed with myocardium or isolated cells from neonatal hearts. NRCM- or NRCF-covered confocal dishes were washed with PBS following diverse treatments and then fixed with 4% paraformaldehyde at 4°C for 30 min. The tissues or cells were then treated with 0.05% Triton X-100 for permeabilization and were incubated with 1% bovine serum albumin, followed by incubation with primary antibodies targeting GPR30 (1:250), MMP-9 (1:800), *p*-ERK (1:800), TGF-β1 (1:200), vimentin (1:250), or troponin Ⅰ (1:100) overnight at 4°C. Then cells were incubated with secondary antibodies for 2 h at room temperature. Nuclear staining was performed with DAPI. Tissue slides and cell slides were examined with an Olympus FV1000 laser confocal microscope (Olympus, Japan). ImageJ software was used to quantify the fluorescence of tissue slides and cell slides in each group.

### Western Blot Analysis

As previously described, protein samples from myocardium and cells were extracted using a RIPA buffer with a protease inhibitor and a phosphatase inhibitor, separated by 8–12% SDS-PAGE, and transferred to a polyvinylidene fluoride membrane (Millipore). Membranes were blocked in Tris-buffered saline with 0.1% Tween containing 5% non-fat milk for 2 h at room temperature, followed by incubation with primary antibodies targeting GPR30 (1:1,000), MMP-9 (1:1,000), *p*-ERK (1:1,000), or TGF-β1 (1:1,000) overnight at 4°C. Then the membranes were incubated with the corresponding secondary horseradish peroxidase–conjugated antibodies (1:5,000) for 2 h at room temperature. The blots were visualized using enhanced chemiluminescence reagent (Millipore). GAPDH was determined as an internal loading control. The density of each band was quantified using Image Lab software (Bio-Rad Laboratories, United States ).

### Determination of MMP-9

The levels of MMP-9 in the culture supernatant of NRCMs in different treatment groups were determined with ELISA kits (Elabscience Bioengineering Institute, China), following the manufacture’s instruction.

### Statistical Analysis

All values were presented as mean ± S.E.M, and all statistical tests were performed using GraphPad Prism software (version 7.0, GraphPad Software Inc, San Diego, CA, United States ). The normality of the distribution was assessed using the Shapiro–Wilk test. The statistical analysis among multiple groups was performed using one-way ANOVA followed by Bonferroni multiple comparisons test. A value of *p* < 0.05 was considered statistically significant.

## Results

### Effects of GPR30 on Cardiac Function and HW/BW in Aged Female Mice Surgery at the Fourth Week After Transverse Aortic Constriction Surgery

**FIGURE 1:**
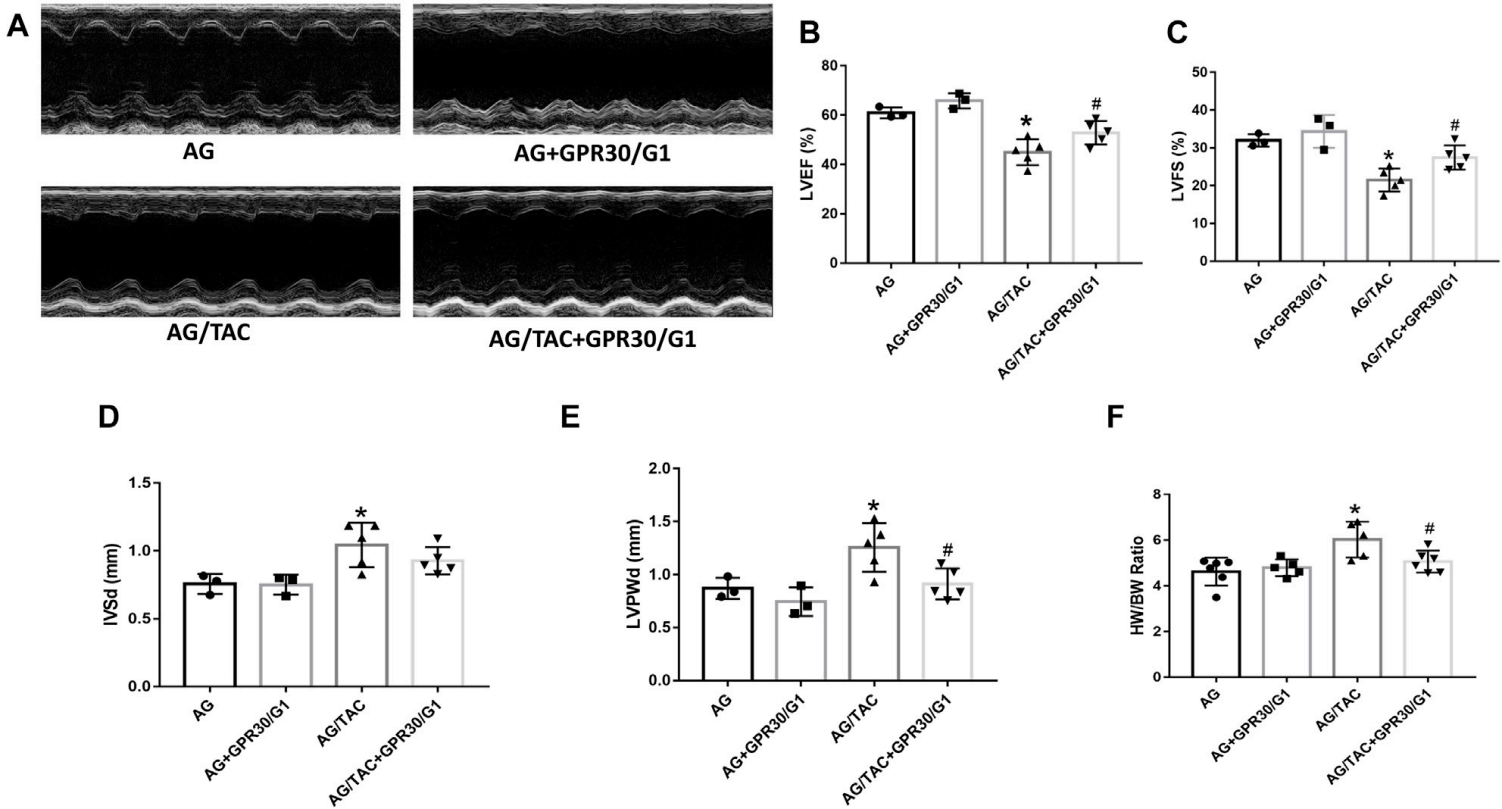
Effects of GPR30 activation on cardiac function four weeks after TAC surgery in aged female mice. Mouse underwent TAC surgery three weeks after tail vein injection of GPR30 adeno-associated virus, and M-mode echocardiography was performed four weeks thereafter. **(A)** Representative M-mode images by echocardiography. **(B)** LVEF. **(C)** LVFS. **(D)** IVSd. **(E)** LVPWd. **(F)** HW/BW ratio. The results were expressed as mean ± S.E.M, *n* = 3–6, *p < 0.05 compared with the AG group. #*p* < 0.05 compared with the AG/TAC group.

This *in vivo* study tried to elucidate the effects of GPR30 activation on cardiac hypertrophy in aged female mice. To fully activate GPR30, the GPR30-specific agonist G1 and GPR30 adeno-associated virus were used. M-mode echocardiography results of different groups are shown in Figure 1. The LVEF and LVFS in the AG/TAC group were worsened than those in the AG group, whereas those in the AG/TAC + GPR30/G1 group were partially recovered compared to those in the AG/TAC group (Figures 1B,C). For cardiac hypertrophy, compared to the AG group, IVSd and LVPWd were significantly enlarged in the AG/TAC group, while the LVPWd in the AG/TAC + GPR30/G1 group was reduced compared to that in the AG/TAC group (Figures 1D,E). The HW/BW ratio of the AG/TAC group was significantly increased compared to that of the AG group, while this ratio was inhibited in the AG/TAC + GPR30/G1 group (Figure 1F). These results together indicated that cardiac function of aged female mice was impaired at the fourth week after TAC surgery, while GPR30/G1 administration could partially recover cardiac function in aged female mice following TAC, showing a cardioprotective effect.

### Effects of GPR30/G1 Treatment on Cardiac Fibrosis of Aged Female Mice at Fourth Week After Transverse Aortic Constriction Surgery

**FIGURE 2:**
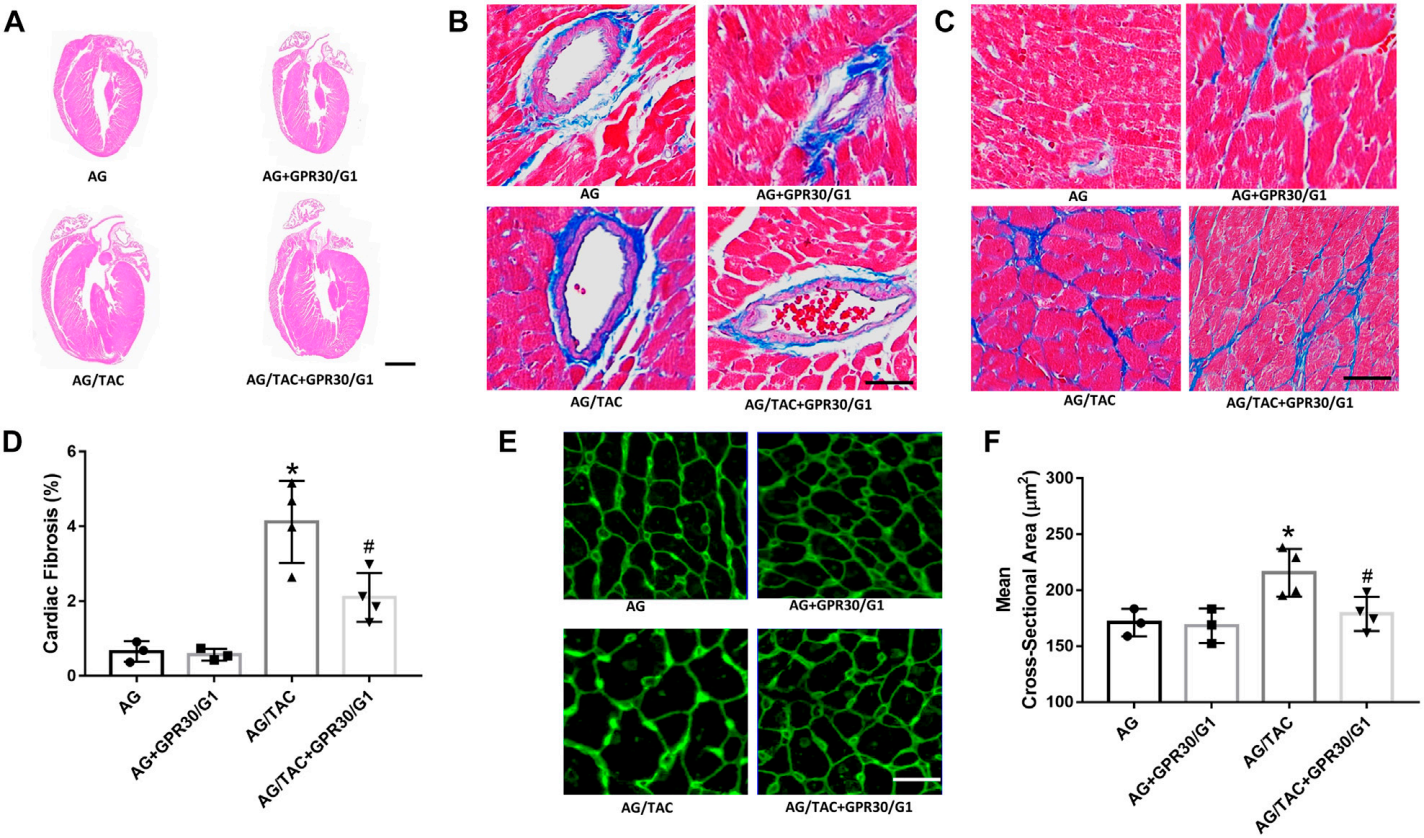
Effects of cardiac fibrosis and morphology four weeks after myocardial TAC surgery in aged female mice. **(A)** Cardiac HE staining (bar = 0.5 mm). **(B–C)** Cardiac Masson trichrome staining (bar = 100 µm). **(D)** Cardiac fibrosis ratio. **(E)** WGA staining (bar = 100 µm). **(F)** Mean cross-sectional area of cardiomyocytes. The results are expressed as mean ± S.E.M, *n* = 3–4. *p < 0.05 compared with the AG group. #*p* < 0.05 compared with the AG/TAC group.

The hallmarks of cardiac remodeling caused by pressure overload are changes in the myocardial cell size, ventricular wall thickness, and myocardial fibrosis. HE staining, Masson staining, and WAG staining were used to observe the effect of GPR30 activation on myocardial remodeling caused by pressure overload at the fourth week after TAC surgery. HE staining showed that the ventricular wall thickness was increased in the AG/TAC group compared to the AG group, while the reduction trend of ventricular wall thickness was observed in the AG/TAC + GPR30/G1 group, which was consistent with our echocardiographic data and the HW/BW ratio. The results of Masson staining (Figures 2B,C) showed that myocardial fibrosis of the heart tissue in the AG/TAC group was significantly elevated compared to the AG group at the fourth week after TAC surgery. GPR30/G1 treatment for 4-week intervention inhibited cardiac fibrosis in the AG/TAC + GPR30/G1 group (Figure 2D). WAG staining results further showed (Figure 2E) that the size of cardiomyocytes in heart tissue in the AG/TAC group was significantly increased compared to the AG group. However, it was significantly reduced after 4-week GPR30/G1 intervention in the AG/TAC + GPR30/G1 group (Figure 2F).

### Effects of GPR30/G1 Treatment on Cardiac Function of Aged Female Mice at Eighth Week After TAC Surgery

**FIGURE 3:**
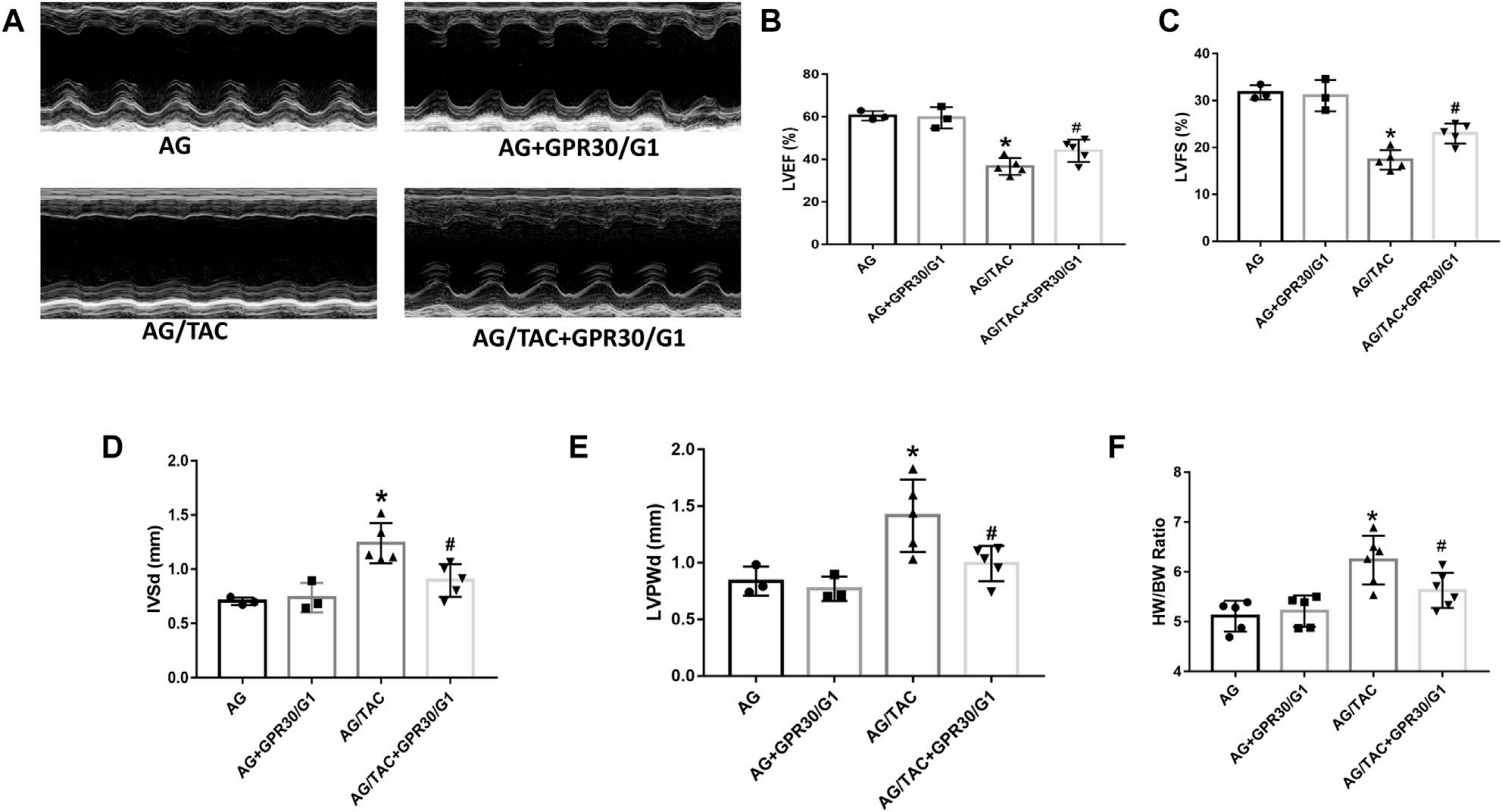
Effects of cardiac function eight weeks after myocardial TAC surgery in aged female mice. Mouse underwent TAC surgery three weeks after tail vein injection of GPR30 adeno-associated virus, and M-mode echocardiography was performed eight weeks thereafter. **(A)** Representative M-mode images by echocardiography. **(B)** LVEF. **(C)** LVFS. **(D)** IVSd. **(E)** LVPWd. **(F)** HW/BW ratio. The results are expressed as mean ± S.E.M, *n* = 3–6, *p < 0.05 compared with the AG group. #*p* < 0.05 compared with the AG/TAC group.

To further assess the long-term effects of GPR30 activation on cardiac hypertrophy in aged female mice, cardiac function and fibrosis were examined in 8 weeks post TAC surgery. The results showed that cardiac LVEF and LVFS in the AG/TAC group further deteriorated compared to those in the AG group, whereas those indices in the AG/TAC + GPR30/G1 group were significantly recovered compared to those in the AG/TAC group (Figures 3B,C). The IVSd and LVPWd in the AG/TAC group were significantly enlarged compared to those in the AG group, whereas those in the AG/TAC + GPR30/G1 group were significantly lower than those in the AG/TAC group (Figures 3D,E). The HW/BW ratio of AG/TAC group was significantly elevated compared to the AG group, while this ratio was greatly decreased in the AG/TAC + GPR30/G1 group (Figure 3F). All these data indicated that the heart function of mice was worsened at the eighth week following TAC surgery, and GPR30/G1 treatment could partially inhibit cardiac damage in the long term in response to pressure overload.

### GPR30/G1 Co-Administration Reduced Cardiac Fibrosis After 8 Weeks of Transverse Aortic Constriction Surgery

**FIGURE 4:**
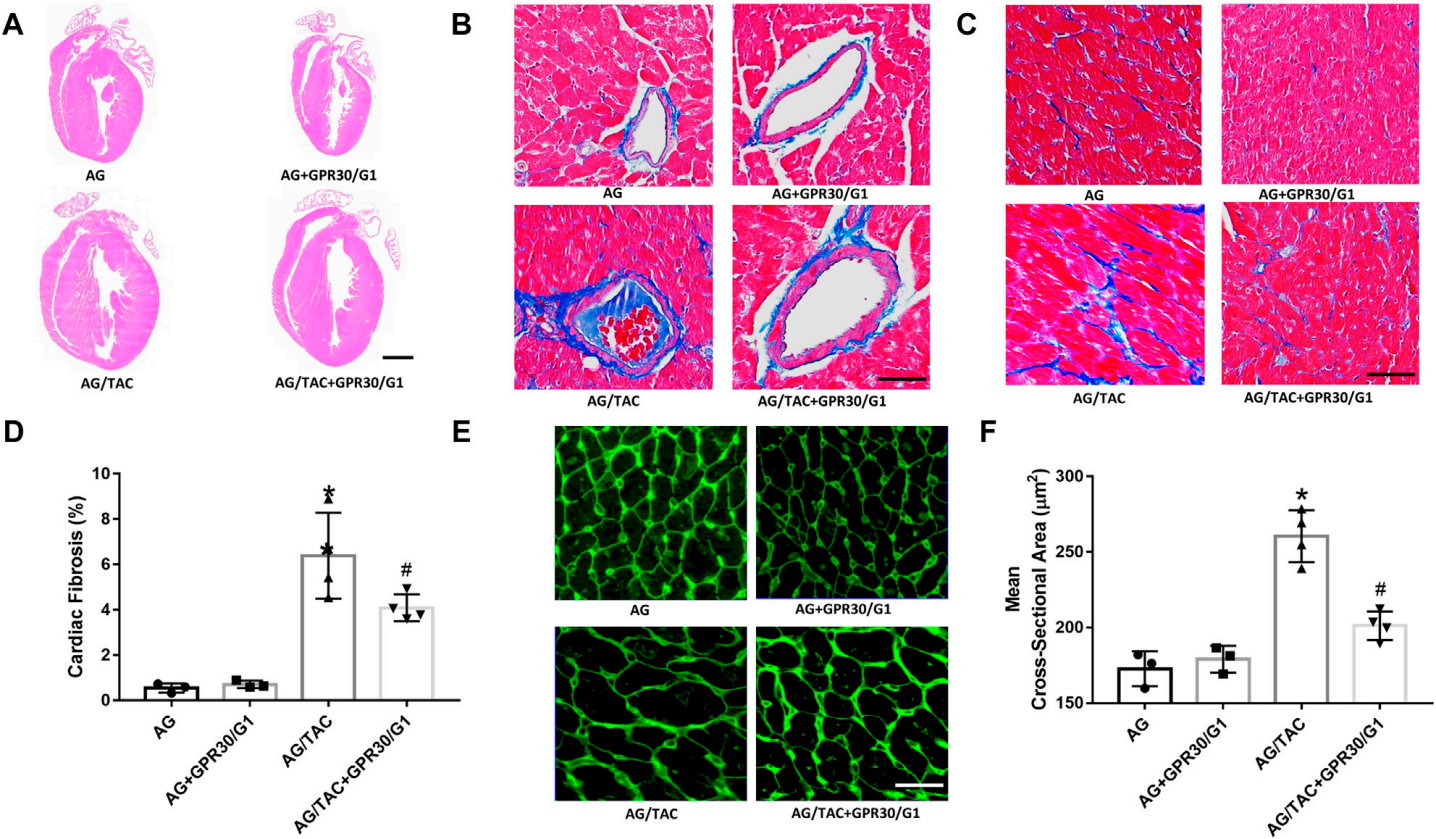
Effects of cardiac fibrosis and morphology eight weeks after myocardial TAC surgery in aged female mice. **(A)** Cardiac HE staining (bar = 0.5 mm). **(B–C)** Cardiac Masson trichrome staining (bar = 100 µm). **(D)** Cardiac fibrosis ratio. **(E)** WGA staining (bar = 100 µm). **(F)** Mean cross-sectional area of cardiomyocytes. The results are expressed as mean ± S.E.M, *n* = 3–4. *p < 0.05 compared with the AG group. #*p* < 0.05 compared with the AG/TAC group.

HE staining, Masson staining, and WAG staining were employed to investigate the effects of GPR30 on cardiac remodeling caused by pressure load after 8 weeks of TAC surgery. The results of Masson staining (Figures 4B,C) showed that myocardial fibrosis in the AG + TAC group was aggravated at the eighth week after TAC surgery compared to the AG group. GPR30/G1 intervention for 8 weeks attenuated cardiac fibrosis in the AG/TAC + GPR30/G1 group (Figure 4D). In order to further confirm whether GPR30/G1 treatment could reduce the cardiac hypertrophy response caused by pressure overload at the time of the eighth week post-TAC surgery, the cross-sectional cell area of cardiac tissue was determined by WGA staining (Figures 4E,F). The average cross-sectional area of cardiac tissue at the time point of the eighth week after TAC surgery in the AG/TAC group was enlarged, while the average myocardial cross-sectional cell area in the AG/TAC + GPR30/G1 group was mitigated following 8 weeks of GPR30/G1 intervention. However, myocardial fibrosis was comparable between the AG group and the AG + GPR30/G1 group.

### Effects of GPR30 Agonist G1 Co-Administration for 8 Weeks on the Expression of GPR30, *p*-ERK, ERK, MMP-9, and TGF-β1 Proteins in Myocardial Tissue of Aged Female Mice in Response to Pressure Overload

**FIGURE 5:**
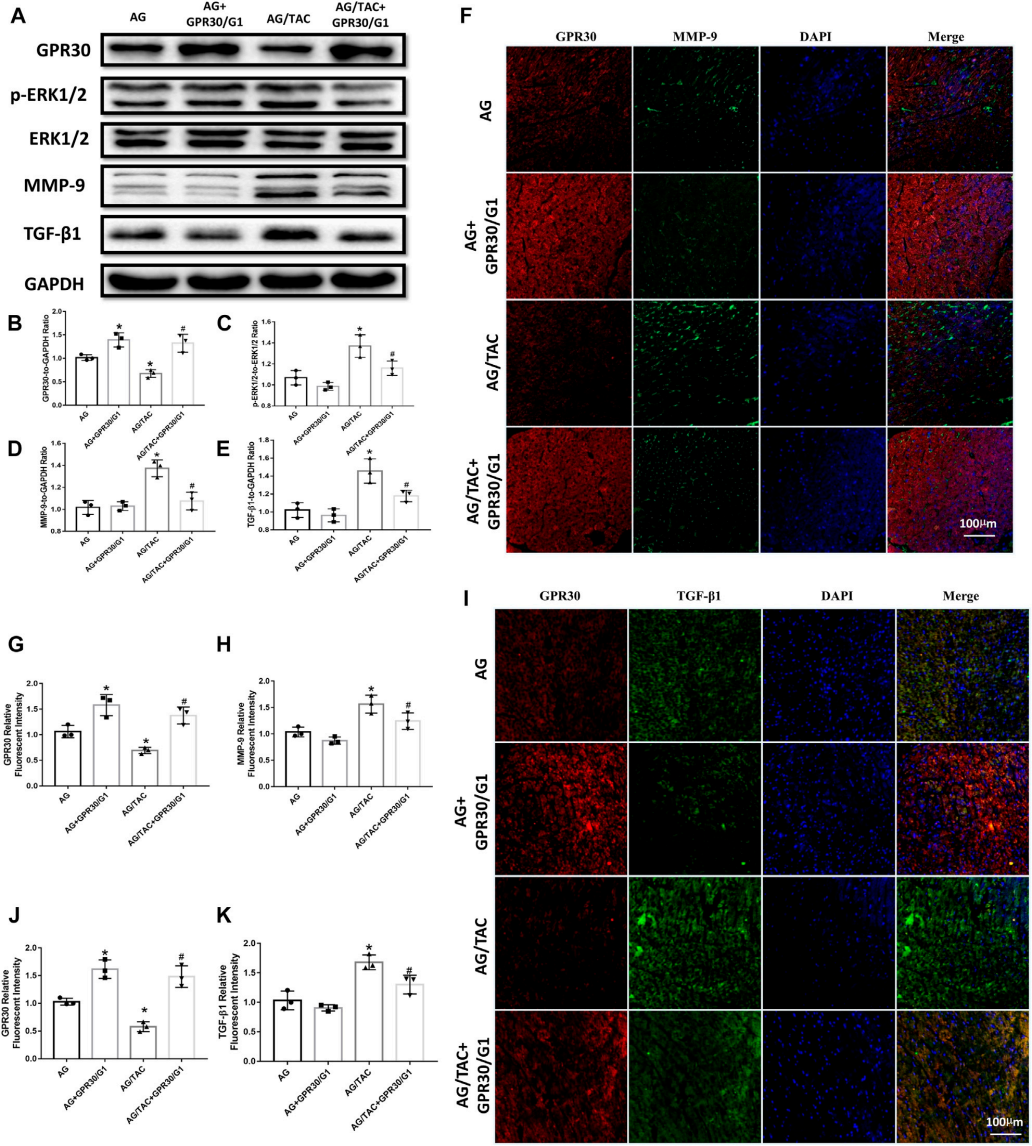
Effects of 8-week GPR30/G1 treatment on the protein expression levels of GPR30, *p*-ERK1/2, ERK1/2, MMP-9, and TGF-β1 in mice subjected to TAC. **(A)** Representative images of the Western blot. **(B)** GPR30 protein level. **(C)** *p*-ERK1/2 to ERK1/2 protein level. **(D)** MMP-9 protein level. **(E)** TGF-β1 protein level. **(F)** Representative confocal microscopy images of myocardial tissue stained with GPR30, MMP-9, and DAPI. Red fluorescence for GPR30 expression. Green fluorescence for MMP-9 expression. Blue fluorescence for nuclei of total cells (bar = 100 μm). **(G)** IFC intensity of GPR30. **(H)** IFC intensity of MMP-9. **(I)** Representative confocal microscopy images of myocardial tissue stained with GPR30, TGF-β1, and DAPI. Red fluorescence for GPR30 expression. Green fluorescence for TGF-β1 expression. Blue fluorescence for nuclei of total cells (bar = 100 μm). **(J)** IFC intensity of GPR30. **(K)** IFC intensity of TGF-β1. The results are expressed as mean ± S.E.M, *n* = 3. *p < 0.05 compared with the AG group. #*p* < 0.05 compared with the AG/TAC group.

Western blot results revealed that continuous intraperitoneal injection of GPR30 agonist G1 for 8 weeks combined with GPR30 overexpression affected the expressions of GPR30, *p*-ERK, ERK, MMP-9, and TGF-β1 proteins in the myocardium of aged TAC mice (Figure 5A). GPR30/G1 significantly increased the expression of GPR30 in the myocardial tissue of the AG + GPR30/G1 group and the AG/TAC + GPR30/G1 group (Figure 5B). Following TAC surgery, the ratio of *p*-ERK1/2 to ERK1/2 in the myocardial tissue of the AG/TAC group increased significantly, while GPR30/G1 can significantly reduce the ratio of *p*-ERK1/2 to ERK1/2 in the myocardial tissue compared to the AG/TAC group (Figure 5C). The expression of MMP-9 in the myocardial tissue of the AG/TAC group was significantly increased, which was significantly decreased by GPR30/G1 treatment (Figure 5D). Meanwhile, the reduction of TGF-β1 expression in myocardium was observed in the AG/TAC + GPR30/G1 group compared to that in the AG/TAC group (Figure 5E).

The results of immunofluorescent staining revealed that GPR30/G1 significantly elevated the expressions of GPR30 in the TAC-injured myocardium of aged female mice (Figures 5F–H). Furthermore, compared with AG, the expression of MMP-9 in the myocardial tissue of the AG/TAC group was significantly increased, while GPR30/G1 intervention decreased the expression of MMP-9 significantly (Figures 5F–H).

As shown in Figure 5 I-K, the expression of TGF-β1 was increased in the AG/TAC group following pressure overload, while GRP30/G1 treatment greatly inhibited the TGF-β1 protein level. Our *in vivo* results showed that GPR30/G1 activation may be associated with the reduction of TGF-β1 and myocardial fibrosis, which may be related with ERK-regulated MMP-9 expression.

### Effects of GPR30 Agonist G1 on the Expressions of *p*-ERK1/2, ERK1/2, and MMP-9 in the Simulated Senescent Cardiomyocytes as well as on the Expression of TGF-β1 in Cardiac Fibroblasts

**FIGURE 6:**
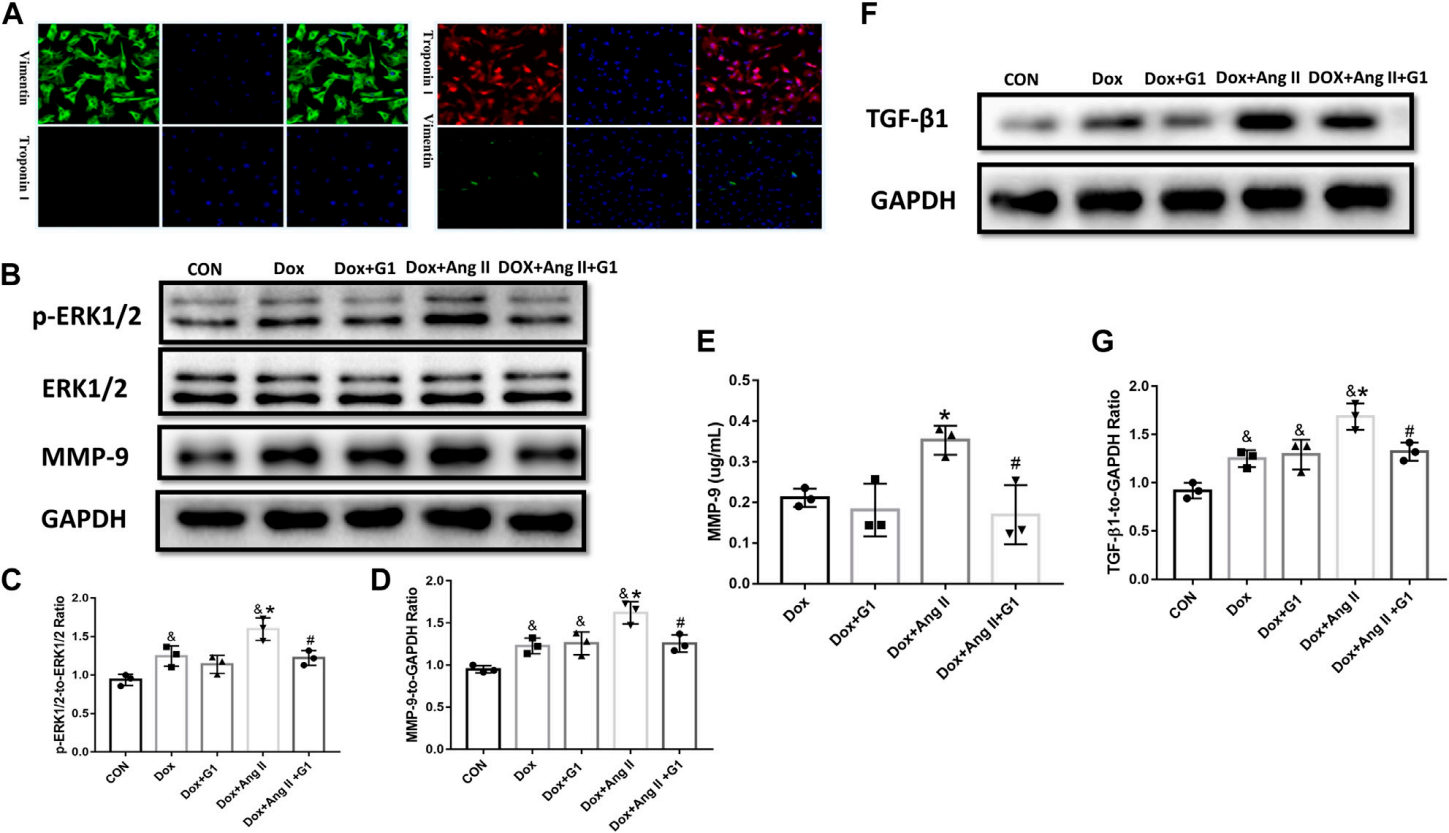
Effects of GPR30 agonist G1 on the expression of *p*-ERK1/2, ERK1/2, and MMP-9 protein in DOX/Ang II-treated cardiomyocytes and on the expression of TGF-β1 in cardiac fibroblast. **(A)** Primary CFs express vimentin and are negative for troponin, which is consistent with the characteristics of primary cultured cardiac fibroblasts. **(B)** Representative images of Western blot. **(C)** *p*-ERK1/2 to ERK1/2. **(D)** MMP-9 expression. **(E)** The content of MMP-9 in the culture medium of cardiomyocytes. **(F)** Representative images of Western blot. **(G)** TGF-β1 expression. The results were expressed as mean ± S.E.M, *n* = 3. *p < 0.05 compared with the Dox group, #*p* < 0.05 compared with the Dox + Ang Ⅱ group.

Primary cultured cardiomyocytes express troponin but not vimentin, and fibroblasts express vimentin but not troponin, which is consistent with the description of cardiomyocytes and cardiac fibroblasts (Figure 6A). Dox and Ang II greatly enhanced the phosphorylated ERK1/2 in cardiomyocytes, while G1 treatment significantly reduced the ratio of *p*-ERK1/2 to ERK1/2 in the Dox group and the Dox + Ang II group (Figure 6 B and C). Furthermore, MMP-9 expression assessed by Western blot and ELISA was significantly inhibited by G1 treatment, which was significantly induced by addition of Dox + Ang II (Figure 6 B, D, and E). Following these treatments, TGF-β1 expression was increased in the Dox + Ang II group, while G1 treatment decreased the expression of TGF-β1 (Figure 6 F and G).

### Effects of GPR30 Antagonist G15 and ERK Inhibitor PD98059 on the Expressions of *p*-ERK1/2, ERK1/2, and MMP-9 in the Simulated Aged Cardiomyocytes as well as on the Expression of TGF-β1 in Cardiac Fibroblasts

**FIGURE 7:**
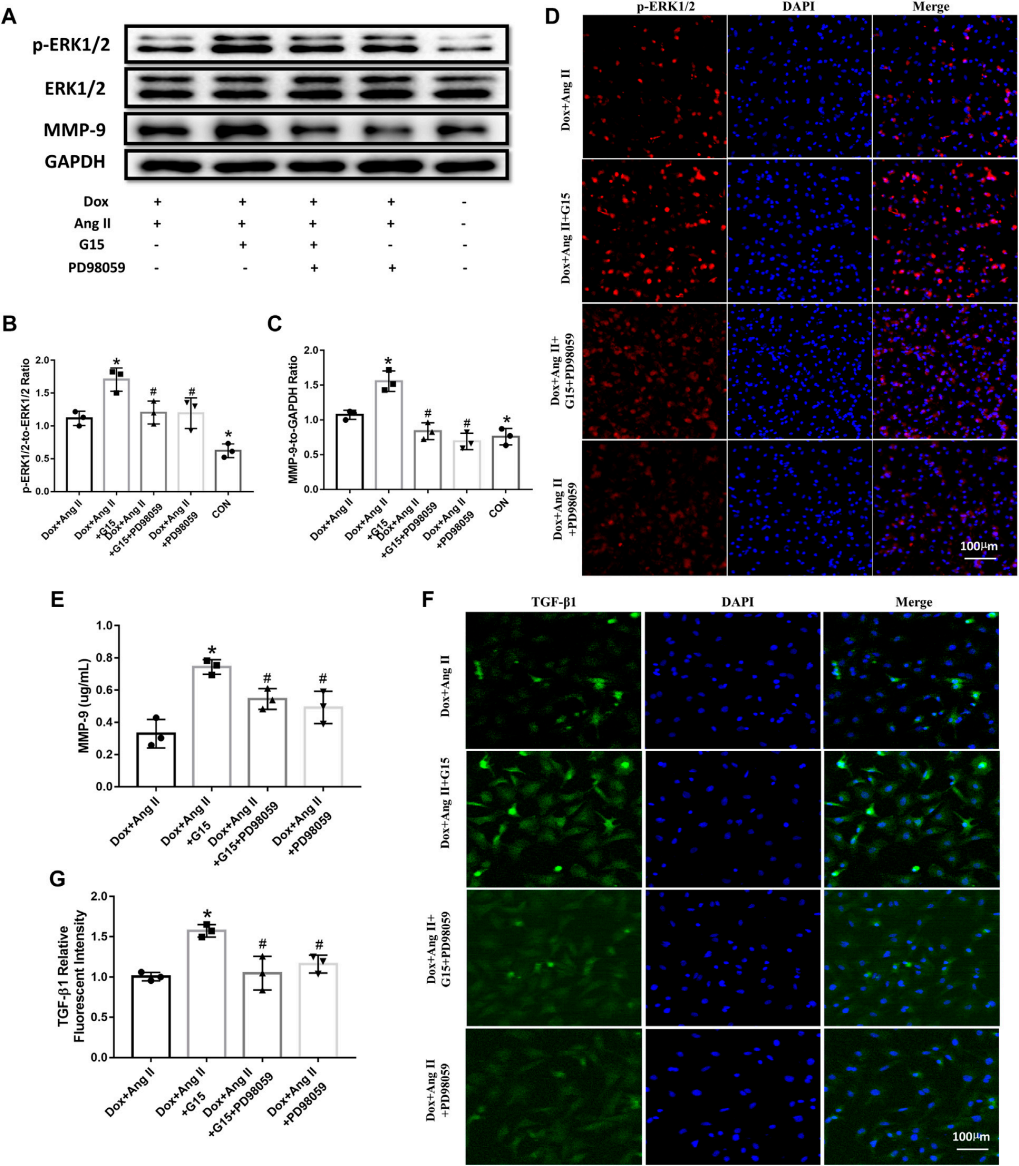
Effects of GPR30 antagonist G15 on *p*-ERK1/2, ERK1/2, and MMP-9 in cardiomyocytes, and the effect on the expression of TGF-β1 in fibroblasts. **(A)** Representative blots of Western blot. **(B)** *p*-ERK1/2 to ERK1/2. **(C)** MMP-9 expression. **(D)** Representative confocal microscopy images of primary cultured cardiomyocytes stained with *p*-ERK and DAPI. Red fluorescence for *p*-ERK expression. Blue fluorescence for nuclei of total cardiomyocytes (bar = 100 μm). **(E)** The content of MMP-9 in the culture medium of cardiomyocytes. **(F)** Representative confocal microscopy images of primary cultured cardiomyocytes stained with TGF-β1 and DAPI. Red fluorescence for TGF-β1 expression. Blue fluorescence for nuclei of total cardiac fibroblasts (bar = 100 μm). **(G)** TGF-β1 expression. *p < 0.05 compared with the Dox + Ang Ⅱ group. #*p* < 0.05 compared with the Dox + Ang Ⅱ+G15 group.

The results from Western blot showed that G15 treatment upregulated phosphorylated ERK1/2 and MMP-9 expression compared to the Dox + Ang II group, while PD98059 could reverse the effects of G15 addition, as evidenced by the reduction of phosphorylated ERK1/2 and MMP-9 expression (Figures 7A–D). The data from ELISA also showed that G15 elevated MMP-9 expression of cell culture medium, while this could be reversed by PD98059 treatment (Figure 7E). Moreover, TGF-β1 expression was increased in the Dox + Ang II + G15 group, while PD98059 treatment decreased the expression of TGF-β1 (Figure 7 F and G).

## Discussion

With the use of ovariectomized rodent models, we have previously explored the function of GPR30 in pathological condition such as myocardial infarction and diabetes-induced myocardial injury (Wang et al., 2018; Wang et al., 2019). Here, we further investigated the effects of GPR30 activation on TAC-induced cardiac hypertrophy of aged female mice. The novel finding of the present study was that GPR30 activation could reduce TAC-induced cardiac fibrosis through downregulation of the MMP-9 level, which may provide the potential therapeutic targets for the treatment of pathological cardiac hypertrophy in postmenopausal women.

Since the incidence of cardiovascular diseases differs significantly between men and women, the protection of estrogen in the cardiovascular system has been proposed and reported for decades (Mendelsohn & Karas, 1999). Especially, the observation of higher risk of cardiovascular diseases with the lower estradiol level among postmenopausal women further emphasized the vital function of estrogen for the cardiovascular system. Thus, several studies including observational research and clinical trials were conducted to evaluate the effects of estrogen supplement on the incidence of cardiovascular diseases of postmenopausal women (Barrett-Connor & Bush, 1991; Rossouw et al., 2002). Although the results from Nurses’ Health Study showed the benefits of estrogen use to decrease both the incidence of coronary heart diseases and the mortality from cardiovascular diseases in menopause women (Stampfer et al., 1991), the reduction of morbidity of cardiovascular diseases by estrogen supplement was not observed in the Framingham Heart Study (Wilson et al., 1985). Therefore, not only double-blind, randomized controlled trials are required to further validate the previous study but also well-designed animal studies should be conducted to reveal the precise molecular mechanisms to better explain these contradictory clinical data.

Estrogen receptors play important roles to mediate multiple biological functions of estrogen. Till date, GPR30 is the only membrane receptor of estrogen that protects the heart from diverse pathological injuries (Mizukami, 2010; Feldman & Limbird, 2017). A previous study showed that the activation of GPR30 by its agonist G1 improved cardiac diastolic function and reduced cardiac fibrosis in ovariectomized female mRen2.Lewis rats (Wang et al., 2012). Moreover, G1 administration attenuated ischemic cardiac injury by using a Langendorff rat model in a gender-independent manner (Deschamps & Murphy, 2009). Our present study employed the aged hypertrophied hearts of female mice to show that LVEF and LVFS in aged female mice were worsened and IVSd and LVPWd were partially recovered at the fourth week post-TAC surgery. The Masson staining revealed that cardiac fibrosis was exacerbated, and the cross-sectional area of cardiomyocytes was further enlarged. Moreover, these indices were aggravated at the eighth week post-TAC. Our results also showed that GPR30 activation could partially recover LVEF and LVFS, inhibit IVSd and LVPWd, and mitigate the fibrotic area of cardiac tissues at both four and eight weeks following TAC surgery. Taken together, these results clearly indicated that activation of GPR30 could partially protect the cardiac function and attenuate cardiac fibrosis in aged female hypertrophied hearts.

Furthermore, the underlying molecular mechanism of GPR30 activation on cardiac fibrosis was further clarified. The present data showed that cardiac fibrotic areas at both myocardial interstitial tissue and the perivascular space were decreased following GPR30 overexpression and G1 administration evidenced by Masson staining. The phosphorylated ERK1/2 was upregulated in response to pressure overload induced by TAC, which as a member of MAPK superfamily has been shown to contribute to pathogenesis of cardiac hypertrophy (Braz et al., 2002; Lorenz et al., 2009). Our data further revealed that GPR30 activation and G1 treatment could inhibit ERK1/2 phosphorylation in aged hypertrophied hearts *in vivo*. These results indicated that this reduction of cardiac fibrosis was associated with suppressed ERK1/2 signaling. Meanwhile, the reduction of MMP-9 in myocardium was observed following GPR30/G1 treatments. The components of the myocardial extracellular matrix were regulated by the balance of numerous matrix metalloproteinases including MMP-9, and the disruption of this balance can cause cardiac fibrosis (Fan et al., 2012). It was reported that MMP-9 expression was induced *via* ERK1/2 signaling in H9c2 cells challenged by lipopolysaccharides (Cheng et al., 2009). Mice lacking MMP-9 have better cardiac function and less left ventricular remodeling than the wild type in response to pressure overload (Heymans et al., 2005), which indicated that MMP-9 inhibitors may preserve cardiac function in pathological hypertrophy. Our results also revealed that MMP-9 expression was increased following TAC surgery in aged female mice hearts, while GPR30 activation could partially inhibit MMP-9 expression. Furthermore, the fibrotic marker protein TGF-β1 was significantly upregulated following TAC surgery in aged female mice hearts, while GPR30/G1 treatment greatly reduced TGF-β1 expression, implying the inhibition of myocardial fibrosis by GPR30 activation. The *in vitro* study showed that G1 treatment decreased angiotensin II and doxorubicin-induced MMP-9 expression in neonatal cardiomyocytes. The MMP-9 level in the supernatant of the cardiomyocyte culture was increased following angiotensin II and doxorubicin treatment, which was reduced by G1 treatment. Furthermore, addition of GPR30 antagonist G15 could elevate ERK1/2 phosphorylation and MMP-9 expression, which can be reversed by ERK1/2 inhibitor PD98059. By adding the culture medium from cardiomyocytes to fibroblasts, the TGF-β1 expression of fibroblasts was increased in the medium, which was treated by angiotensin II and doxorubicin, while the TGF-β1 expression of fibroblast was inhibited in the medium which was treated by G1. Taken together, these results favored the notion that GRP30 activation inhibited MMP-9 expression *via* ERK1/2 signaling to at least partially preserve cardiac function and inhibited myocardial fibrosis in aged female hypertrophied hearts.

Although the ovariectomized female mouse is a powerful model for studying the effects of estrogen use, aging is a more complex pathological process, instead of bilateral oophorectomy. Thus, in order to fully mimic the pathological condition of postmenopausal women, mice at the age of 18 months were used in this study. Second, despite overexpression of GPR30 by adeno-associated virus delivery in our study, we further intraperitoneally injected the agonist G1 to thoroughly activate GPR30. While our present study clarified the vital role of GPR30 activation in aged hypertrophied female hearts, there are some limitations, which can be improved in further studies. In this study, we used GPR30 adeno-associated virus to overexpress GPR30. But GPR30 knockout or transgenic mice should be used in future studies, the result of which may provide valid evidence. Second, we only observed cardiac hypertrophy and cardiac function till 8 weeks post-TAC surgery. The long-term results may indicate the potential beneficial or detrimental effects of GPR30 activation.

In summary, this study demonstrated that GPR30 and G1 co-administration reduced TAC-induced cardiac fibrosis and preserved cardiac contractile function in aged female hearts. These effects were attributed to GPR30 activation and the subsequent inhibition of ERK1/2-mediated MMP-9 expression. By using an *in vitro* model, the importance of ERK1/2 in mediating GPR30 protection against TAC-induced cardiac fibrosis was verified by ERK1/2 signaling inhibitor PD98059. Collectively, our results presented the new potential drug for the treatment of cardiac pathological hypertrophy in postmenopausal women.

## Data Availability

The raw data supporting the conclusion of this article will be made available by the authors, without undue reservation.
