# Supplementary figures and images for: G Protein–Coupled Estrogen Receptor 30 Reduces Transverse Aortic Constriction–Induced Myocardial Fibrosis in Aged Female Mice by Inhibiting the ERK1/2 -MMP-9 Signaling Pathway
Source: Front Pharmacol. 2021 Nov 5;12:731609. doi: 10.3389/fphar.2021.731609 (PMC8603421; doi:10.3389/fphar.2021.731609)

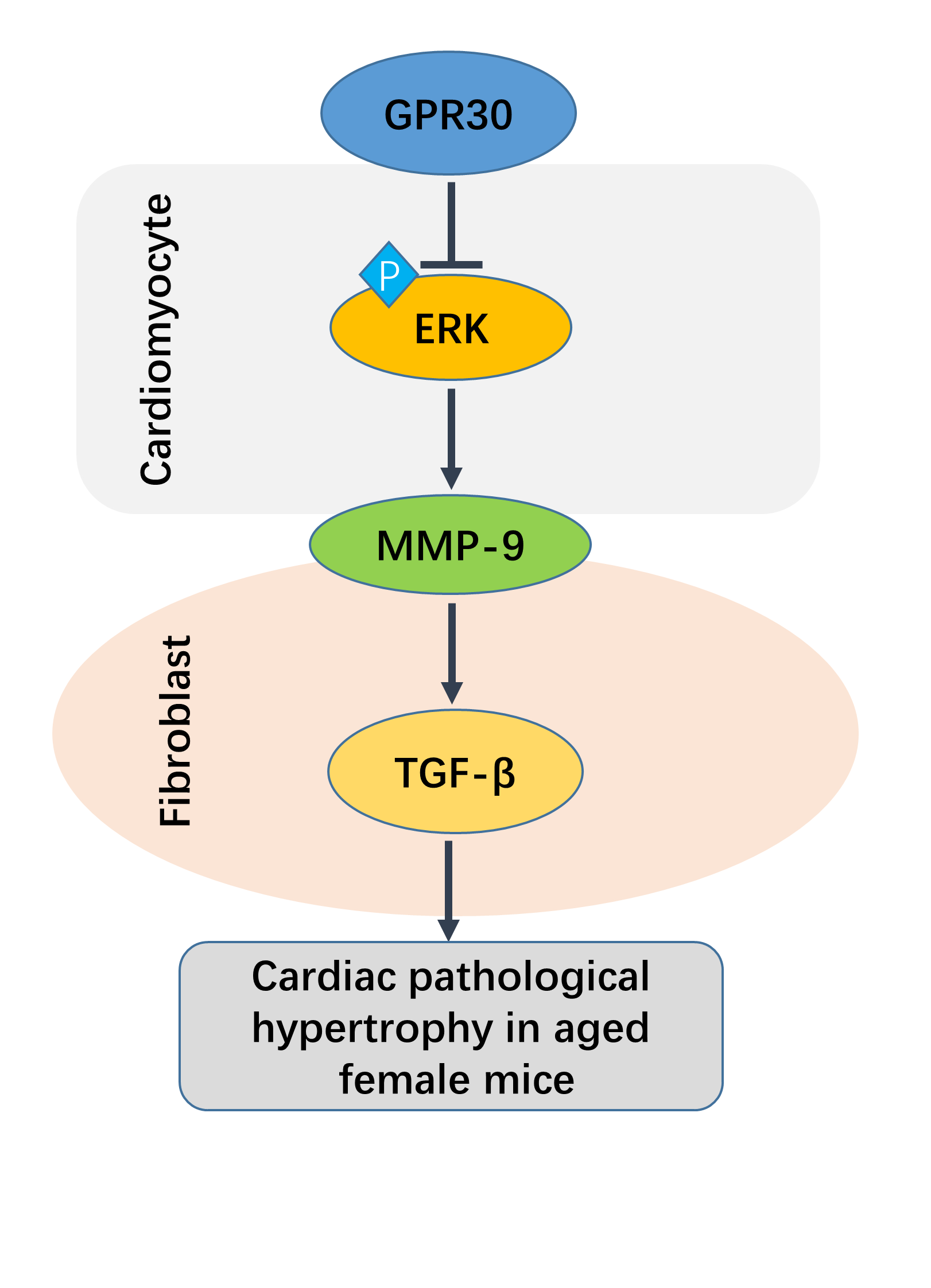

Supplement: Supplementary file 1 [file Image1.TIF]
