# Supplementary material for: G Protein–Coupled Estrogen Receptor 30 Reduces Transverse Aortic Constriction–Induced Myocardial Fibrosis in Aged Female Mice by Inhibiting the ERK1/2 -MMP-9 Signaling Pathway
Source: Front Pharmacol. 2021 Nov 5;12:731609. doi: 10.3389/fphar.2021.731609 (PMC8603421; doi:10.3389/fphar.2021.731609)
Supplement: Supplementary file 2 [file DataSheet1.docx]

**S. Fig. 1**


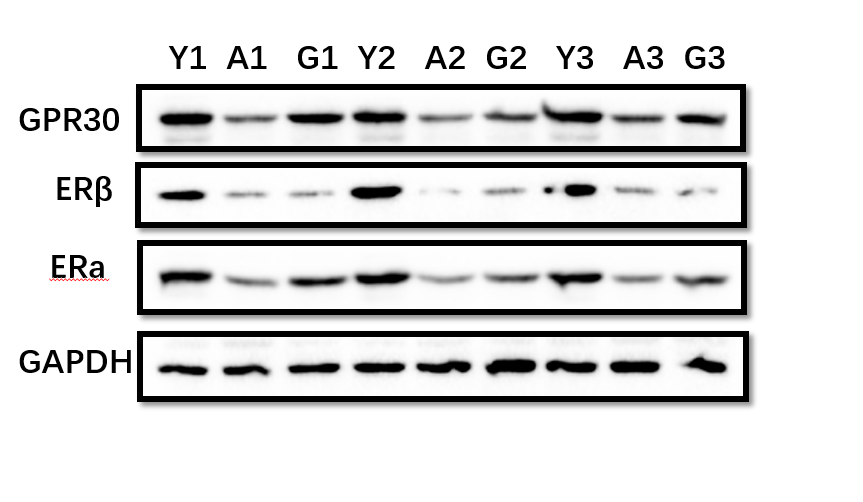


S Fig. 1 The estrogen receptors expressions among young female mice, aged female mice and aged female mice with GPR30/G1 treatment. N=3. Y, young female mice; A. aged female mice; G, aged female mice with GPR30/G1 treatment.

**S. Fig. 2**


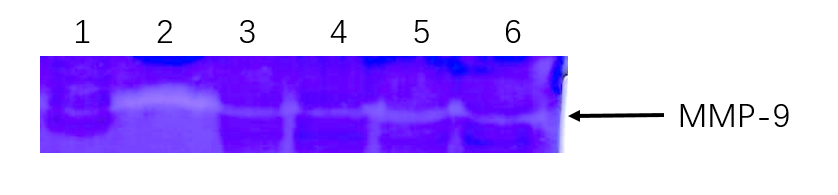


S Fig. 2 The MMP-9 activity measurements of tissue lysis from mouse heart following treatments. The activity was assessed by zymography following the manufacture’s instruction (Xin Fan biotech). 1, Protein Maker; 2, positive control (mouse blood plasma); 3, aged female mice; 4, aged female mice with GPR30/G1 treatment; 5, aged female mice with TAC surgery; 6, aged female mice with TAC surgery and GPR30/G1 treatment.
